# Predicting prolonged sick leave among trauma survivors

**DOI:** 10.1038/s41598-018-37289-w

**Published:** 2019-01-11

**Authors:** Erik von Oelreich, Mikael Eriksson, Olof Brattström, Andrea Discacciati, Lovisa Strömmer, Anders Oldner, Emma Larsson

**Affiliations:** 1 Perioperative Medicine and Intensive Care, Karolinska University Hospital, Solna, Stockholm, Sweden; 2 Section of Anaesthesiology and Intensive Care Medicine, Department of Physiology and Pharmacology, Karolinska Institute, Stockholm, Sweden; 3 Unit of Biostatistics, Institute of Environmental Medicine, Karolinska Institute, Stockholm, Sweden; 4 Division of Surgery, Department of Clinical Science, Intervention and Technology (CLINTEC), Karolinska Institute, Stockholm, Sweden

## Abstract

Many survivors after trauma suffer from long-term morbidity. The aim of this observational cohort study was to develop a prognostic prediction tool for early assessment of full-time sick leave one year after trauma. Potential predictors were assessed combining individuals from a trauma register with national health registers. Two models were developed using logistic regression and stepwise backward elimination. 4458 individuals were included out of which 488 were on sick leave full-time 12 months after the trauma. One comprehensive and one simplified model were developed including nine and seven predictors respectively. Both models showed excellent discrimination (AUC 0.81). The comprehensive model had very good calibration, and the simplified model good calibration. Prediction models can be used to assess post-trauma sick leave using injury-related variables as well as factors not related to the trauma *per se*. Among included variables, pre-injury sick leave was the single most important predictor for full-time sick leave one year after trauma. These models could facilitate a more efficient use of resources, targeting groups for follow-up interventions to improve outcome. External validation is necessary in order to evaluate generalizability.

## Introduction

Trauma is a major cause of mortality and morbidity; globally more than five million people die due to injuries every year^1^. The number of victims left with significant disabilities is several-fold higher^2^. In Sweden, trauma is the most common cause of death for individuals under the age of 45^3^. The relatively young age of trauma patients leads to significant losses to society in terms of human life years, sustained morbidity and costs. Recently, our group investigated sustained long-term morbidity for trauma patients using sick leave as a proxy^4^. The study demonstrated that apart from variables related to injuries, also factors not related to the trauma *per se* were associated with long-term sick leave. Furthermore, it is well recognized that long-term disability and morbidity after severe trauma is hard to measure and even harder to predict^5^. Resources in most health care systems are scarce and it is unmanageable to thoroughly follow all individuals for an extended period of time. Even though there is an association between several trauma and non-trauma factors and sick leave, a prediction model could be an important practical tool for identification of patient groups with a high risk of long-term sick leave after trauma. Such a tool could be a useful instrument to implement a more efficient use of limited resources targeting groups for interventions to improve outcome.

The aim of the study was to develop and validate a prognostic prediction model for assessment of long-term sick leave. In addition, a second aim was to develop a simplified, more feasible model and evaluate its performance compared to the more comprehensive model. Our hypothesis was that a simplified model would perform less well.

## Methods

### Setting and study population

A single centre retrospective observational cohort study of trauma patients admitted to the Karolinska University Hospital, a level-one trauma centre in Stockholm, was conducted. The development cohort comprised all admissions from January 2005 until December 2010. All patients suffering severe physical trauma admitted with trauma team activation, regardless of Injury Severity Score (ISS), as well as individuals admitted without trauma team activation, but later found to have an ISS > 9 are included in a trauma register. Patients with isolated fractures of the upper or lower extremity, drowning, chronic subdural hematomas, burn injuries and hypothermia without concomitant trauma are not included in the register.

Individuals between 20 and 63 years were included in the study, younger individuals were excluded since few of them are expected to work full time. In Sweden, the retirement age is generally 65 years, therefore 63 years was set as the upper limit for inclusion. Individuals receiving 100% disability pension at the time of trauma were excluded, since they are not entitled to sick leave in Sweden. Students and unemployed are also entitled to financially compensated sick leave, and were thus included in the study. Individuals without a valid personal identification number were excluded since information on pre-trauma exposure was not available in national registers. All individuals were followed for 12 months. The study adhered to the Transparent Reporting of a multivariable prediction model for Individual Prognosis Or Diagnosis (TRIPOD statement) for prediction models^6^.

### National registers

The unique Swedish personal identification number enables linkages between different national registers^7^. The National Patient Register, managed by the National Board of Health and Welfare (NBHW), covers information on public inpatient and outpatient care not classified as primary care^8^. Each care episode is classified according to the International Classification of Diseases (ICD-10). NBHW also manages the Cause of Death Register that records individual data on time and cause of death. Statistics Sweden is responsible for statistics concerning the national census and administers the Total Population Register.

### Assessment of outcome

The main outcome of the prediction model was full-time sick leave the 12^th^ month after the trauma. Outcome data were extracted from the Social Insurance Agency. Disability pension provided after the trauma was considered equal to sick leave.

### Assessment of predictors

Sick leave the month before trauma was divided into none, part-time and full-time sick leave. Data on comorbidity was extracted from the National Patient Register and retrieved up to eight years prior to trauma. Somatic comorbidity was defined as the presence of any of the somatic diagnoses included in the Charlson Comorbidity Index modified to ICD-10^9^. Psychiatric comorbidity was defined as the presence of a diagnosis in ICD-10 groups F20-F99 and substance abuse as a diagnosis in F10-F19 respectively. The Longitudinal Integration Database for Health Insurance and Labour Market Studies (LISA) managed by Statistics Sweden provided data on education categorised as low, medium or high, representing ≤9 years (elementary school only), 10–12 years (senior high school) and >12 years (equal to university level). Data on injury characteristics were extracted from the trauma register. Injury severity was classified according to the ISS based on the Abbreviated Injury Scale (AIS) 1990 edition for year 2005–2006 and AIS 2005 edition for year 2007–2010.

### Selection of potential predictors

**Figure 1:**
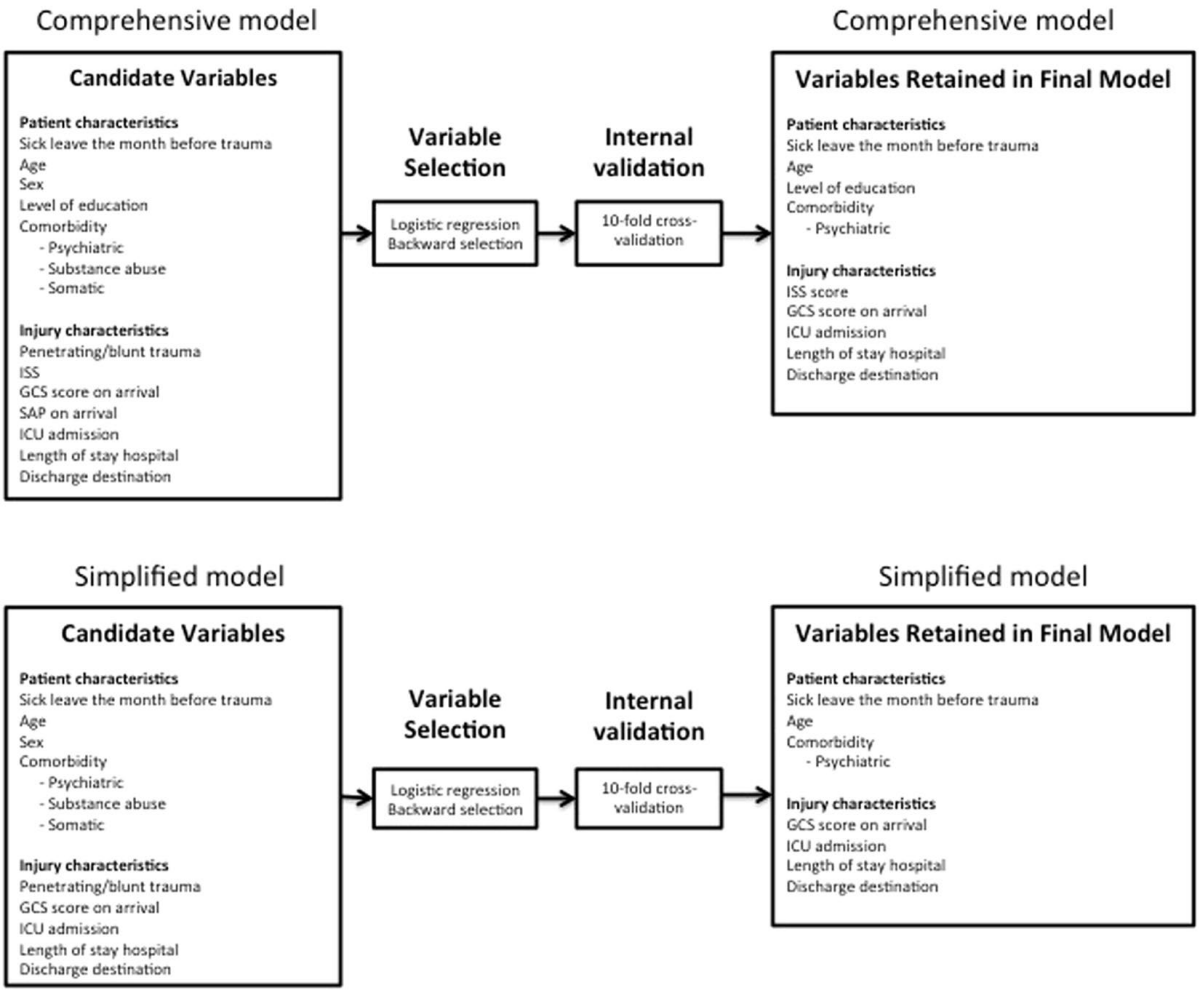
Model building for the comprehensive and the simplified model.

The candidate variables for modelling sick leave after trauma were chosen based on data availability, physiological plausibility and the existing literature^4,5,10–17^. Seven patient-related and seven trauma-related variables were considered for inclusion in the comprehensive prediction model, all considered to be reproducible in various trauma settings (Fig. 1).

The second objective was to investigate how well a simplified model would perform compared to the initial, more comprehensive model. Out of all parameters in the comprehensive model, we selected eleven predictors accessible at discharge (Fig. 1).

### Statistics

By using logistic regression, two prognostic models based on the two sets of candidate predictors were generated. The predictor variables included in the models were selected using a backward selection algorithm with a significance level for removal from the model set to 0.2^18^.

The discrimination and the calibration of the model selection procedure was internally validated using 10-fold cross-validation^19^. With this technique, the study population is randomly divided into ten groups of equal size (deciles). Then, model selection using the aforementioned backward algorithm is performed on nine of the ten deciles (training data) and the resulting model is used for prediction on the remaining decile (validation data). This process is repeated ten times, such that each decile of the data is used for validation exactly once.

The area under the operating characteristic curve (AUC) statistic calculated using the cross-validated predicted probabilities was used to assess model discrimination – that is separating people with full-time sick leave one year after trauma from people without full-time sick leave. An AUC between 0.7 and 0.8 was considered to indicate acceptable discrimination, between 0.8 and 0.9 excellent discrimination, and above 0.9 outstanding discrimination^20^.

Calibration – that is agreement between observed and predicted risk – was assessed by means of calibration slope and calibration plot^21^. The calibration slope was estimated by fitting a logistic regression to the study outcome, including the logit transform of the cross-validated predicted probabilities as the only covariate^18^. Ideally, the calibration slope is equal to 1. Moderate calibration was graphically assessed by plotting the mean predicted probabilities versus the observed proportions by decile of prediction. Ideally, the observed proportions are equal to the predicted probabilities, resulting in the plotted data points being aligned with the graph’s diagonal.

Predicted probabilities of full-time sick leave 12 months after the trauma can be calculated using the following formula:

$${\rm{p}}=1/(1+{{\rm{e}}}^{-{\rm{xb}}})$$

where

xb = *β*_0_ + ∑*βi* × *x*_*i*_, *β* refers to the regression coefficients, and *x* to the selected predictors. Formulas to compute the linear predictor xb, and therefore the predicted probabilities, are reported in the Results section for the two prognostic models.

In order to facilitate the use of the two prognostic models, we graphically presented them using nomograms^22,23^. Nomograms provide a straightforward way of calculating probabilities of full-time sick leave one year after trauma without the need of a computer or a calculator^24^. The nomograms are provided in the supplement material. All analyses were performed using Stata/MP release 14.2 (StataCorp, College Station, TX).

### Ethics

The study was approved by the regional ethical review board in Stockholm, Sweden (approval numbers 2008/249-31/3, 2009/862-32 and 2011/1705-32). No patient consent was needed for a retrospective register-based study according to the ethical approval. All research was performed in accordance with national guidelines and regulations.

## Results

**Figure 2:**
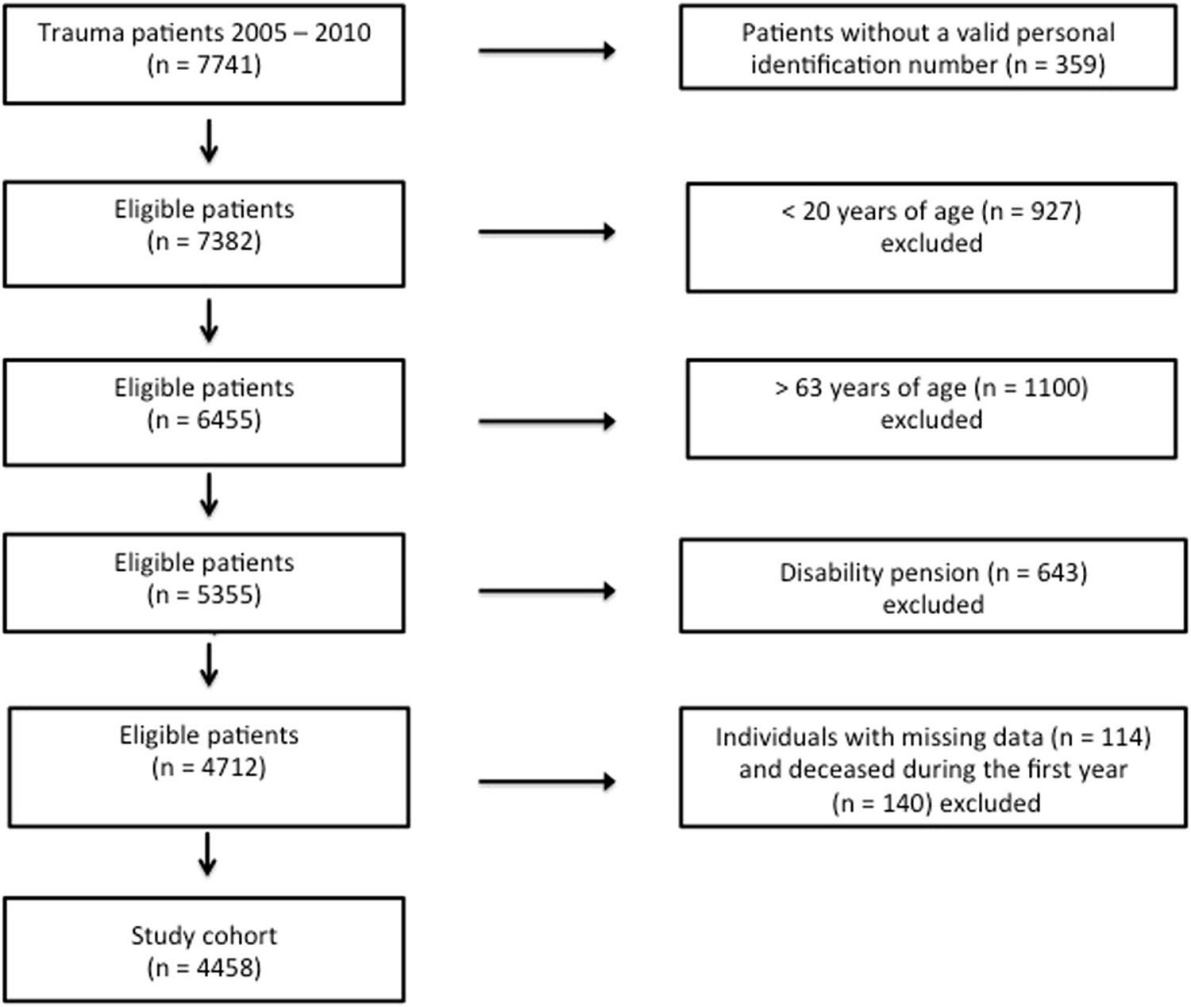
Flow chart of included patients.

Individuals with missing data on education (n = 114) and individuals deceased during the first year following trauma (n = 140) were excluded (Fig. 2).

### Patient characteristics

The 4458 individuals in the final study cohort included trauma patients from the greater Stockholm region with a first trauma admission between 2005 and 2010. In total 488 individuals were on full-time sick leave the 12^th^ month after the trauma. Demographic and trauma characteristics of the study cohort are presented in Table 1 and Table 2. Potential differences based on sick leave status the 12^th^ month after the trauma were investigated using the chi-square test. Individuals with full-time sick leave after the trauma were older, had more comorbidities (psychiatric, somatic and substance abuse) and more sick leave the month before trauma compared to individuals without the outcome. In addition, the former group was more seriously injured with higher ISS, had a lower Glasgow Coma Scale score (GCS) on arrival, were treated in the Intensive Care Unit (ICU) to a greater extent, had longer hospital length of stay and fewer were discharged directly home.

**Table 1:** Patient characteristics, description of potential predictors by outcome.

Variable	Not full-time sick leave the 12^th^ month after the trauma	Full-time sick leave the 12^th^ month after the trauma	p-value*
n (%)	3970	488	
Sick leave the month before trauma			<0.001
None	3825 (96.3)	375 (76.8)	
Part	109 (2.7)	71 (14.5)	
Full	36 (0.9)	42 (8.6)	
Age			<0.001
20–29	1352 (34.1)	100 (20.5)	
30–44	1411 (35.5)	190 (38.9)	
45–63	1207 (30.4)	198 (40.6)	
Sex			0.095
Female	1144 (28.8)	123 (25.2)	
Male	2826 (71.2)	365 (74.8)	
Comorbidity			
Psychiatric	392 (9.9)	95 (19.5)	<0.001
Substance abuse	468 (11.8)	94 (19.3)	<0.001
Somatic	594 (15.0)	100 (20.5)	0.001
Level of education			0.010
Low	928 (23.4)	136 (27.9)	
Medium	1979 (49.8)	249 (51.0)	
High	1063 (26.8)	103 (21.1)	

Values in parentheses are percentages unless indicated otherwise. *chi-square test.

**Table 2:** Injury characteristics, description of potential predictors by outcome.

Variable	Not full-time sick leave the 12^th^ month after the trauma	Full-time sick leave the 12^th^ month after the trauma	p-value*
n (%)	3970	488	
Type of trauma			0.19
Penetrating	256 (6.4)	24 (4.9)	
Blunt	3714 (93.6)	464 (95.1)	
ISS			<0.001
≤15	3331 (83.9)	261 (53.5)	
16–24	431 (10.9)	92 (18.9)	
25–40	177 (4.5)	105 (21.5)	
>40	31 (0.8)	30 (6.1)	
GCS			<0.001
14–15	3553 (89.5)	320 (65.6)	
9–13	232 (5.8)	56 (11.5)	
3–8	185 (4.7)	112 (23.0)	
SAP			<0.001
≥90 mm Hg	3920 (98.7)	468 (95.9)	
<90 mm Hg	50 (1.3)	20 (4.1)	
ICU admission			<0.001
Yes	738 (18.6)	253 (51.8)	
No	3232 (81.4)	235 (48.2)	
Length of stay hospital			<0.001
0–7 days	3330 (83.9)	221 (45.3)	
>7 days	640 (16.1)	267 (54.7)	
Discharge destination			<0.001
Home	3311 (83.4)	216 (44.3)	
Other hospital/rehab	659 (16.6)	272 (55.7)	

Values in parentheses are percentages unless indicated otherwise. Injury Severity Score (ISS). Glasgow Coma Scale (GCS). Systolic Arterial Pressure (SAP). Millimetres of mercury (mm Hg). Intensive Care Unit (ICU). *chi-square test

### The comprehensive model

**Figure 3:**
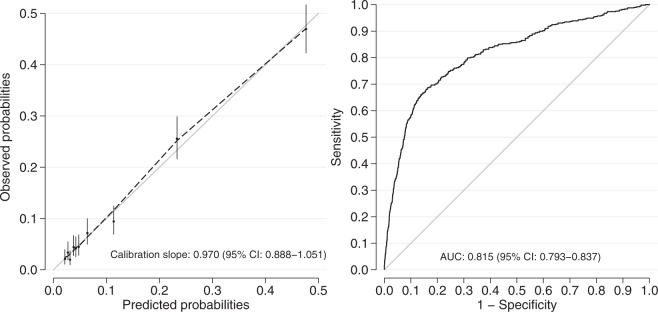
Calibration curve comparing observed and predicted risks for full-time sick leave the 12^th^ month after the trauma and area under the Receiver Operating Curve of the comprehensive predictive model.

The final comprehensive prediction model for sick leave one year after trauma included nine predictors: age, sick leave the month before trauma, psychiatric comorbidity, education level, ISS, GCS-score on arrival, ICU-admission, length of stay at hospital, and discharge destination. The predictors are presented with odds ratios (OR) and 95% corresponding confidence intervals (CI) in Table 3. The performance of the comprehensive model presented as the area under the receiver operating characteristic curve (AUC) was 0.81, which was regarded as excellent discrimination. The expected versus actual risk for full-time sick leave the 12^th^ month after the trauma, showed a very good calibration (calibration slope (95% CI): 0.970 (0.888–1.051)) (Fig. 3). To calculate the predicted probabilities for the comprehensive model as explained in the Methods section, the following formula to calculate the linear predictor xb is used: xb = −4.0218 + 2.0431 (if on part-time sick leave the month before trauma) + 2.4163 (if on full-time sick leave the month before trauma) + 0.5756 (if age 30 to 44) + 0.5971 (if age 45 to 63) + 0.3724 (if psychiatric comorbidities are present) + 0.3086 (if medium level of education) + 0.5326 (if high level of education) − 0.0279 (if ISS 16 to 24) + 0.5389 (if ISS 25 to 40) + 0.3580 (if ISS above 40) + 0.2274 (if GCS 9 to 13) + 0.6142 (if GCS 3 to 8) + 0.5010 (if admitted to ICU) + 0.9167 (if more than 7 days of hospital length of stay) + 0.8073 (if discharged to other hospital or rehabilitation centre).

**Table 3:** Final comprehensive model for full-time sick leave the 12^th^ month after the trauma. Injury Severity Score (ISS). Glasgow Coma Scale (GCS). Intensive Care Unit (ICU).

Variable	Regression coefficient	OR (95% CI)	p-value
Sick leave the month before trauma			
None	0		
Part	2.0431	7.71 (5.31–11.21)	<0.001
Full	2.4163	11.20 (6.60–19.02)	<0.001
Age			
20–29	0		
30–44	0.5756	1.78 (1.34–2.36)	<0.001
45–63	0.5971	1.82 (1.36–2.42)	<0.001
Comorbidity			
Psychiatric	0.3724	1.45 (1.06–1.98)	0.019
Level of education			
High	0		
Medium	0.3086	1.36 (1.03–1.79)	0.028
Low	0.5326	1.70 (1.25–2.32)	0.001
ISS			
≤15	0		
16–24	−0.0279	0.97 (0.70–1.36)	0.87
25–40	0.5389	1.71 (1.19–2.47)	0.004
>40	0.358	1.43 (0.76–2.69)	0.27
GCS			
14–15	0		
9–13	0.2274	1.26 (0.87–1.82)	0.23
3–8	0.6142	1.85 (1.31–2.61)	<0.001
ICU admission			
No	0		
Yes	0.501	1.65 (1.23–2.22)	0.001
Length of stay hospital			
0–7 days	0		
>7 days	0.9167	2.50 (1.83–3.41)	<0.001
Discharge destination			
Home	0	0	
Other hospital/rehab	0.8073	2.24 (1.69–2.97)	<0.001
Constant	−4.0218

### The simplified model

**Figure 4:**
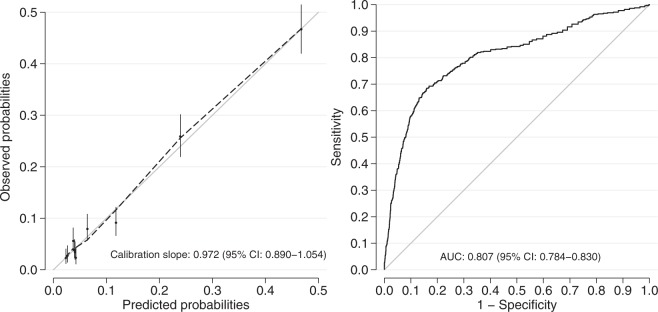
Calibration curve comparing observed and predicted risks for full-time sick leave the 12^th^ month after the trauma and area under the Receiver Operating Curve of the simplified predictive model.

In the final simplified model seven predictors were included; age, sick leave the month before trauma, psychiatric comorbidity, GCS-score on arrival, ICU-admission, length of stay at hospital and discharge destination. The predictors are presented with OR and 95% CI in Table 4. The AUC of the simplified model was 0.81, which was regarded as excellent discrimination. The graphical presentation of expected versus actual risk for the outcome indicated a good calibration (calibration slope (95% CI): 0.972 (0.890–1.054)) (Fig. 4). The formula for the linear predictor xb is: xb = −3.6813 + 2.0925 (if on part-time sick leave the month before trauma) + 2.4526 (if on full-time sick leave the month before trauma) + 0.5050 (if age 30 to 44) + 0.5063 (if age 45 to 63) + 0.3476 (if psychiatric comorbidities are present) + 0.2882 (if GCS 9 to 13) + 0.6921 (if GCS 3 to 8) + 0.5679 (if admitted to ICU) + 0.9743 (if more than 7 days of hospital length of stay) + 0.8611 (if discharged to other hospital or rehabilitation centre).

**Table 4:** Final simplified model for full-time sick leave the 12^th^ month after the trauma.

Variable	Regression coefficient	OR (95% CI)	p-value
Sick leave the month before trauma			
None	0		
Part	2.0925	8.10 (5.59–11.76)	<0.001
Full	2.4526	11.62 (6.87–19.65)	<0.001
Age			
20–29	0		
30–44	0.505	1.66 (1.25–2.19)	<0.001
45–63	0.5063	1.66 (1.25–2.20)	<0.001
Comorbidity			
Psychiatric	0.3476	1.42 (1.04–1.93)	0.028
GCS			
14–15	0		
9–13	0.2882	1.33 (0.92–1.93)	0.13
3–8	0.6921	2.00 (1.43–2.79)	<0.001
ICU admission			
No	0		
Yes	0.5679	1.76 (1.33–2.35)	<0.001
Length of stay hospital			
0–7 days	0		
>7 days	0.9743	2.65 (1.97–3.56)	<0.001
Discharge destination			
Home	0		
Other hospital/rehab	0.8611	2.37 (1.80–3.11)	<0.001
Constant	−3.6813		

Glasgow Coma Scale (GCS). Intensive Care Unit (ICU).

## Discussion

In this cohort study we developed a prognostic prediction model assessing post-trauma sick leave. A comprehensive model including nine parameters was derived and validated. In our trauma cohort, this model predicted the risk for prolonged sick leave after trauma with high precision. Given the complexity of this model, we compared its performance with a simplified version including seven parameters. As expected, the simplified model performed less well but demonstrated a surprisingly high precision. After external validation, these models might serve as useful tools to assess and identify long-term morbidity after trauma, measured as sick leave.

It is complex to fully assess the complete burden of trauma. Current data indicate that for every trauma fatality, between three and four patients survive with a serious or permanent disability^25^. Thus, apart from death, there is a significant suffering for patients and relatives considering the vast number of trauma victims. Moreover, these victims are relatively young. As of today, a significant proportion of trauma patients end up with long-term morbidity.

In the literature, mortality is often used as the primary endpoint after trauma, whereas the number of studies investigating long-term outcomes in terms of morbidity is limited^26^. In an Australian study, return to work was used as a proxy for morbidity, with high numbers of disability two years after trauma^5^. In a recent study published by our group, sick leave was used as a proxy for morbidity with similar findings – sick leave rates did not return to pre-injury levels during the 36 month follow-up period^4^. It has been proposed that sick leave might be a measure of morbidity in a population and may serve as a summary measure of physical, psychological and social functioning^27^. Therefore, a practical tool identifying risk groups could be a valuable improvement when allocating scarce resources.

When developing the initial, more comprehensive model, parameters associated with the trauma *per se* as well as patient characteristics were assessed in the variable selection process (Fig. 1). The candidate variables were chosen based partly on the results from a previous association study investigating prolonged sick leave after trauma^4^, data availability in different registers, and physiological plausibility.

Among the nine parameters in the final model, the inclusion of ISS and GCS-score was somewhat expected since serious injuries and reduced consciousness are associated with conditions with significant residual long-term morbidity^28,29^. ICU stay might be considered to reflect the physiological response to the trauma including the significance of comorbidities, the injury severity and the need for special monitoring. The inclusion of this predictor is in line with the results from Dinh *et al*. who described that ICU admission is associated with a need for rehabilitation after trauma^30^. Hospital length of stay and discharge destination might also reflect overall injury severity, although it is important to emphasize that these parameters obviously are affected by other components and differences in health care systems.

The non trauma-related variables, psychiatric disease and level of education, were also included in the comprehensive model. We have previously demonstrated that psychiatric comorbidity and low socioeconomic positions are associated with an increased risk of both trauma and sick leave^4,31^. The fact that the only comorbidity included in the prediction model was psychiatric disease is somewhat surprising^5^. It has been suggested that the variables age and comorbid conditions to some extent carry the same information^9^, hence the inclusion of only one of these variables in the final model. The strongest predictor was pre-injury sick leave. Sick leave might serve as a proxy for pre-injury overall morbidity in the trauma population explaining its relative importance. Overall the comprehensive model demonstrated very good calibration and excellent discrimination.

Our second objective was to derive a simplified prediction model and to evaluate its precision. The reason for this was the fact that some of the predictors included in the comprehensive model, for example ISS, may not be accessible at discharge, since they require involvement of the registrars responsible for the trauma register. Therefore, we decided to compare our initial comprehensive model with a simplified version only including variables easy to obtain already at discharge. All parameters were assumed easy to extract from the patient’s medical record or of such nature that any patient would be able to give a correct answer. Again, trauma-related as well as patient-related parameters were included (Fig. 1). The simplified model did not perform as well as the comprehensive model. However, this model was surprisingly accurate with a good calibration and excellent discrimination. The main advantage of the simplified model is the possibility for a more practical use early accessible at discharge.

A possible future aspect of this study, after external validation, is to try to identify what *type* of post-trauma morbidity individuals with full-time sick leave suffer in order to initiate appropriate measures.

The use of sick leave as a proxy for post-trauma morbidity can be questioned. Clearly individuals without significant morbidity may be on sick leave and vice versa depending on social factors and type of occupation. However, there is a clear association between morbidity and sick leave justifying its use as a proxy for estimation of morbidity in a large cohort where the alternatives are elaborate and sparse^27^. A general limitation includes the retrospective register-based design. The single centre approach limits the generalizability since demographics, injury types and treatments might differ in other settings. Difficulties in transferring a prediction model to other populations are well known, mostly due to the lack of external validity. However, it is likely to assume that the possible differences in populations comparing the Stockholm region with other regions in Scandinavia and several European countries are relatively small. The format of the existing social security system in different countries will most likely influence the main outcome, full-time sick leave the 12^th^ month after the trauma. Emigration during the study period could influence loss to follow up. However, we have no reason to believe that there is a difference between exposed (trauma patients) and unexposed (uninjured patients). The annual national emigration rate was less than 0.5% during the study period. Strengths of the study include the large size of the trauma cohort as well as the wide range of examined variables including both trauma-related parameters and pre-existing conditions. The final variables included in our models are routinely available across other trauma centres increasing the possibility to perform a future external validation. A very low rate of missing data and minimal loss to follow-up also strengthen the study. Regardless of system, sick leave is most likely associated with post-trauma morbidity and could be considered one way of describing sustained morbidity. Finally, the use of 10-fold cross-validation to assess internal validation and overfitting improves the quality of the study.

In this single centre retrospective observational cohort study we developed two different prediction models identifying individuals at risk for prolonged sick leave after trauma. Injury-related factors as well as factors not related to the trauma *per se* were important predictors for the study endpoint, full-time sick leave the 12^th^ month after the trauma. A comprehensive model with high precision including nine parameters was derived and internally validated. Furthermore, a simplified model including seven predictors, all accessible at hospital discharge, was also developed. This simplified model was less accurate, but still with good enough precision. These prediction models may serve as tools for identification of patient groups with a high risk of sick leave after trauma and could facilitate a targeted use of follow-up resources. External validation is necessary in order to evaluate generalizability.

## Supplementary information

Supplementary material

## Data Availability

The data that support the findings of this study are available from Trauma Registry Karolinska and national registers. Restrictions apply to the availability of these data, which were used under license for the current study, and so are not publicly available. Data is however available from the authors upon reasonable request and with permission of Trauma Registry Karolinska and the NBHW.
