# Supplementary material for: Predicting prolonged sick leave among trauma survivors
Source: Sci Rep. 2019 Jan 11;9:58. doi: 10.1038/s41598-018-37289-w (PMC6329751; doi:10.1038/s41598-018-37289-w)
Supplement: Supplementary file 1 — Supplementary material [file 41598_2018_37289_MOESM1_ESM.docx]

**Predicting prolonged sick leave among trauma survivors**

**Authors**

Erik von Oelreich* (EvO)^1,2^ MD, Mikael Eriksson (ME)^1,2^ MD, Olof Brattström (OB)^1,2^ MD, PhD, Andrea Discacciati (AD)^3^ PhD, Lovisa Strömmer (LS)^4^ MD, PhD, Anders Oldner (AO)^1,2^ MD, PhD and Emma Larsson (EL)^1,2^ MD, PhD.

(erik.vonoelreich@sll.se, mikael.er.eriksson@sll.se, olof.brattstrom@sll.se, andrea.discacciati@ki.se, lovisa.strommer@sll.se, anders.oldner@ki.se, emma.ca.larsson@sll.se)

*Nomograms*

In Figure 5, a nomogram for the comprehensive model is shown. This is interpreted by adding the score for all variables; for example an individual aged 25 with no history of psychiatric comorbidity, low level of education and part-time sick leave before the trauma who suffers from a trauma resulting in a GCS 9 on arrival, ISS of 30, admitted to the ICU, a hospital stay of seven days and discharged to home. Age 25 and no psychiatric comorbidity both correspond to a score of 0; low level of education approximately to a score of 2.2; part-time sick leave to a score of 8.5; GCS 9 to a score of 1; ISS 30 to a score of 2.4; ICU-admission to a score of 2; hospital stay of seven days to a score of 0 and finally discharge to home to a score of 0. This adds up to a total score of 16.1, which means that this patient has approximately a 45 % probability of full-time sick leave the 12^th^ month after the trauma.

**

*Figure 5. Nomogram for the comprehensive model*

In Figure 6, a nomogram for the simplified model is presented, interpreted in the same way as for the comprehensive model described above.

**

*Figure 6. Nomogram for the simplified model*

**Figure legends**

*Figure 5. Nomogram for the comprehensive model*

Nomogram for prediction of full-time sick leave the 12^th^ month after the trauma using the comprehensive model.

*Figure 6. Nomogram for the simplified model*

Nomogram for prediction of full-time sick leave the 12^th^ month after the trauma using the simplified model.
